# RNASeq Analysis of the Shoot Apex of Flax (*Linum usitatissimum*) to Identify Phloem Fiber Specification Genes

**DOI:** 10.3389/fpls.2016.00950

**Published:** 2016-06-28

**Authors:** Ningyu Zhang, Michael K. Deyholos

**Affiliations:** ^1^Department of Biological Sciences, University of AlbertaEdmonton, AB, Canada; ^2^Department of Biology, University of British ColumbiaKelowna, BC, Canada

**Keywords:** meristem, RNASeq, vascular differentiation, fiber, xylem, phloem

## Introduction

All of the post-embryonic, above-ground structures of seed plants are generated from the shoot apical meristem (SAM), which acts as a reservoir of stem cells. Members of the flax genus (*Linum* spp.) have been used historically as models for the study of SAMs (Esau, 1942). Cultivated flax (*Linum usitatissimum*) is grown in more than 50 countries for its seeds or its stem phloem (bast) fibers (Rubilar et al., 2010). Due to prolonged intrusive growth, and a highly crystalline cellulosic secondary wall, flax phloem fibers are among the longest and strongest cells in plants (Mohanty et al., 2000). In flax, all phloem fibers are derived from primary growth in the shoot apex. Specification of phloem fibers occurs in the apical-most 0.5 mm of the stem, since young phloem fibers can be anatomically distinguished starting 0.4–0.5 mm from the shoot apex (Gorshkova et al., 2003). The molecular mechanisms that govern fiber identity are almost entirely unknown (Gorshkova et al., 2012). Also, in contrast to the significant progress obtained in the past decade toward understanding xylem differentiation, information about the phloem fiber differentiation is very scarce (De Rybel et al., 2016). In the past decade, shoot apex transcriptomes have been described in various plants, including maize, pea, soybean, rice, Arabidopsis and chickpea, but none of these produce significant primary phloem fibers (Ohtsu et al., 2007; Wong et al., 2008; Haerizadeh et al., 2009; Jiao et al., 2009; Yadav et al., 2009; Wang et al., 2014). Most molecular and cellular research on flax fiber has thus far focused on later stages of development (Day et al., 2005; Roach and Deyholos, 2007; Fenart et al., 2010). Differential transcript expression data from the region of the shoot apex in which fiber specification occurs would complement other approaches (e.g., mutant screening) aimed at understanding primary phloem fiber differentiation.

## Value of the data

- The genetic basis of primary phloem fiber identity in any species is unknown. This limits basic research and crop improvement.
- Data were obtained from tissues at high spatial resolution, which allows the results to be correlated with specific developmental processes.
- The identification of transcripts enriched in the shoot apical region will help define mechanisms of phloem fiber specification, and contribute to improved understanding of the SAM in general.

## Data

We used RNAseq to compare transcript expression patterns in two segments of the vegetative stem of 14d flax plants, from which all visible leaves had been removed. The segments were: (i) the apical region (AR) of the shoot apex, which contained the apical-most 0.5 mm of the stem, including the SAM and its immediate derivatives; and (ii) the basal region (BR), which contained the entire stem except for the apical-most 1 cm, and therefore represented all stem and vascular tissues at later stages of differentiation as compared to the AR. Four biologically independent replicates of AR (AR1, AR2, AR3, AR4), and two biologically independent replicates of BR (BR1, BR2) were sequenced on the Illumina HiSeq platform in a total of nine runs. Data were deposited in the Sequence Read Archive (SRA) as the following accessions: AR1: SRR1056618; AR2: SRR1056620, SRR1056621; AR3: SRR1056622, SRR1056623; AR4: SRR1056624, SRR1056625; BR1: SRR1038482; BR2: SRR1421513 (http://www.ncbi.nlm.nih.gov/sra?term=SRP033325). In total, 117.5 million clean reads (21 Gbp) were obtained and mapped to the reference flax genome to detect transcripts enriched in the AR as compared to the BR. These differential transcript expression data (measured as normalized FPKM) are available at NCBI GEO (GSE80718; http://www.ncbi.nlm.nih.gov/geo/query/acc.cgi?acc=GSE80718), and an annotated version of the file is available as Supplemental Table 1.

## Experimental design, materials, and methods

### Plant materials

**Figure 1:**
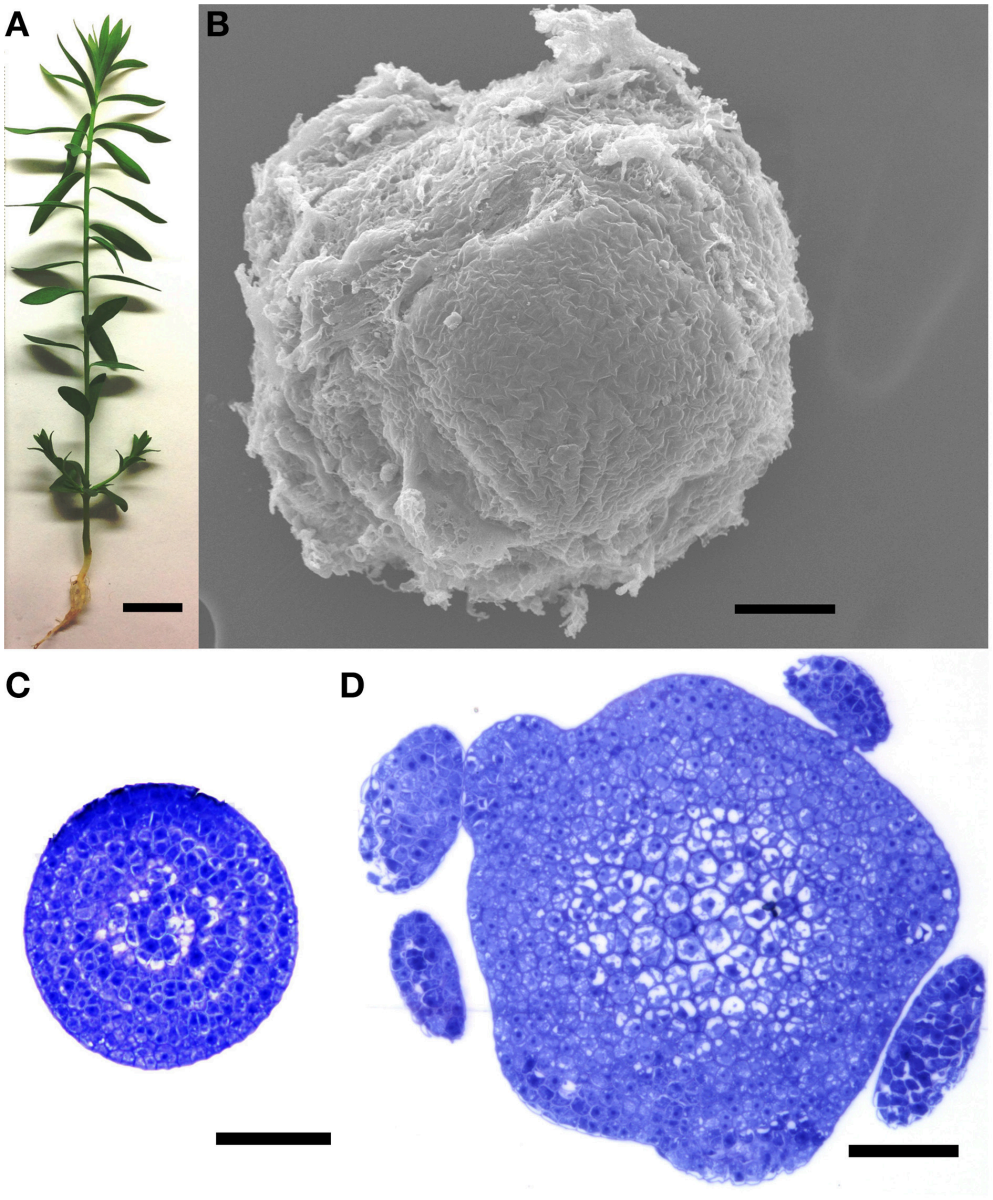
**Plant tissues used for library construction. (A)** A 14 day plant at the time of dissection. **(B)** Environmental scanning electron micrograph of an unfixed, dissected shoot apical region (AR), representative of the tissue used for RNA extractions. **(C,D)** Transverse sections through the apical **(C)** and basal **(D)** limits of the shoot apical region (AR), showing extent of morphological differentiation at time of RNA extraction. Plants used for RNA extraction did not contain the leaf primordia seen in **(D)**. Scale bars **(A)** 1 cm; **(B–D)** 50 μm.

Flax (i.e., linseed) plants (*L. usitatissimum* L. cv. CDC Bethune; Rowland et al., 2002) were grown in potting mix in an environmental chamber at 22°C, with a cycle of 16 h light and 8 h dark, as previously described (Wang et al., 2012). Fourteen days after germination (Figure 1A), approximately 0.5 mm of the apical-most part of each stem (the apical region, AR) was dissected under a Leica S6D stereo microscope, all visible leaf primordia were removed, and the tissue was frozen in liquid nitrogen. A representative dissection, visualized under an environmental scanning electron microscope, is shown in Figure 1B, and transverse sections of a shoot apex, corresponding to the apical and basal-most tissues sampled, are shown in Figures 1C,D. Shoot apices were similarly dissected from approximately 200 plants and pooled prior to each RNA extraction. After collecting the shoot apex, the remainder of the stem (i.e., the basal region, BR) from 1 cm below the shot apex to the stem base was also dissected, stripped of leaves, visible lateral branches and axillary meristems, and frozen in liquid nitrogen. In this way, maturing stems from at least six plants were pooled for each RNA extraction. For RNASeq of the AR, samples were harvested from four biological replicates (i.e., four sets of plants that were grown spatially and temporally independently from each other), and tissues were obtained from two biologically independent replicates were used for the BR. For qRT-PCR, three additional, independent biological replicates (i.e., different plants than those used for RNASeq) were obtained from each of the AR and BR.

### RNA extraction and sequencing

RNA from each biological replicate (Section Plant Materials) was extracted separately. RNeasy Micro Kit (Qiagen) and RNeasy Plant Mini Kit (Qiagen) were used to isolate RNA from the AR and BR samples, respectively. Extracted RNA was then digested with TURBO DNA-free™ Kit (Life Technologies) to remove DNA contamination and their quality was evaluated using a RNA 6000 Nano chip (Agilent Technologies) on an Agilent 2100 Bioanalyzer. Total RNA was delivered to the service provider, BGI (Shenzen, China), where each biological replicate was sequenced separately. oligodT coupled magnetic beads were used to isolate poly-A+ mRNA, which was used as a template for cDNA synthesis (Superscript II, Invitrogen) primed by random hexamers, followed by second strand synthesis using *E. coli* DNA PolI (Invitrogen). Double-stranded cDNA (Qiaquick PCR Purification Kit, Qiagen), was sheared with a nebulizer, end repaired, and ligated to Illumina PE adapter oligos, and the products size-selected by gel purification to produce 200 bp fragments. These were PCR amplified through 15 cycles to prior to sequencing using an Illumina HiSeq 2000 with 90 bp, paired-end reads. The quality of the sample during processing prior to sequencing was monitored using the Agilent 2100 Bioanalyzer and ABI StepOnePlus Real-Time PCR System. Because the sequencing output for samples AR2, AR3, and AR4 was slightly lower than expected (9.6 million reads output per sample), additional aliquots of each of these three samples were sequenced in three additional runs. Raw reads from all runs were filtered to remove adapter sequences, contamination, and low-quality reads, and the filtered raw reads were deposited in the SRA archive. Each of the nine paired read files were uploaded to SRA in fastq format.

### RNASeq analysis of differential transcript abundance

To quantify the relative abundance of transcripts in the shoot apex (AR) as compared to the remainder of the stem (BR), the clean sequencing reads described in Section RNA Extraction and Sequencing were mapped to the flax reference genome (Wang et al., 2012; downloaded from Phytozome 9 as Lusitatissimum_200.fa) using Tophat2 (Trapnell et al., 2012), and the accepted hits were used as input for cufflinks, with default parameters. The resulting assemblies were merged and with the reference genome annotation (downloaded from Phytozome 9 as Lusitatissimum_200_gene.gff3) with cuffmerge, and finally Cuffdiff was used to calculate normalized differential transcript abundance between the samples. The output of cuffdiff (gene_exp.diff) is available at NCBI GEO as accession GSE80718, and an annotated version of this file is available as Supplemental Table 1. The merged.gtf file is available as Supplemental Table 2, and defines positions of a locus identified by cufflinks, in reference to the scaffolds in the Lusitatissimum_200.fa genome assembly. Within these results, transcripts for 6207 genes were significantly (*q* < 0.05) more abundant in AR compared to BR, and 4405 of these were enriched at least 2-fold in the AR. Conversely, transcripts for 8388 genes were significantly (*q* < 0.05) more abundant in BR compared to AR and 7901 of these were enriched at least 2-fold in the BR. Inspection of the data showed that several markers of shoot apex tissues were highly enriched in the AR sample. For example, *PROTODERMAL FACTOR 1* (*PDF1*) transcripts have been reported to be expressed exclusively in the L1 layer of meristems and the protoderm of organ primordia (Abe et al., 1999). In our results, transcripts of putative *PDF1* genes (Lus10007351, Lus10031390, Lus10010941) were at least 19.5-fold more abundant in AR than BR (Supplemental Table 1). Similarly, *CUP-SHAPED COTYLEDON* (*CUC*) genes are required for SAM function and organ separation (Hasson et al., 2011). Transcripts of three putative *CUC* genes (Lus10041924, Lus10005537, Lus10013205) were at least 45-fold more abundant in AR than BR; two other putative CUC genes (Lus10037106, Lus10003458) were not detected in either sample. As a third example, the SHOOT MERISTEMLESS (STM) transcription factor is essential for SAM formation and maintenance (Endrizzi et al., 1996); a putative STM gene (Lus10030003) was 4.8-fold enriched in the AR sample compared to BR. Conversely, several markers of late differentiation were more enriched in the BR compared to the AR. For example, *CELLULOSE SYNTHASE A* (*CESA*) genes *CESA4, CESA7*, and *CESA8* are associated with secondary wall synthesis (Chantreau et al., 2015); we observed transcripts of flax genes annotated as *CESA4* (Lus10008225, Lus10008226), and *CESA8* (Lus10007296, Lus10029245) to be at least 125-fold enriched in the BR compared to the AR (no CESA7 genes were identified in the original flax genome annotation used in this study). Another well-established marker of xylem differentiation, *XYLEM CYSTEINE PROTEINASE-2* (*XCP2*; Avci et al., 2008). The two putative flax *XCP2* genes (Lus10030722, Lus10013204) were enriched 106-fold in the BR compared to the AR. Thus, expression of at least some well-known markers of early and late stem development were observed in patterns that matched expectations.

### Quantitative real-time PCR analysis of differential transcript abundance

**Figure 2:**
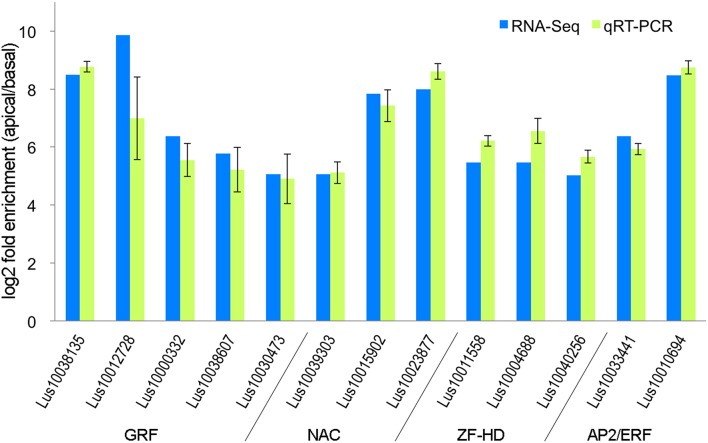
**Ratio of transcript abundance in the stem apical region (AR) compared to the basal region (BR), as measured by qRT-PCR and RNA-Seq on independently grown tissues**.

To evaluate the accuracy of the differential transcript expression measurements that we obtained (Section RNASeq Analysis of Differential Transcript Abundance) we used qRT-PCR to measure transcript abundance in independently grown replicates of the same tissues that were used for RNA-Seq. In order to select an appropriate reference gene for the qRT-PCR, GeNorm was used to determine the expression stability of nine commonly used reference genes among tissues assayed in our study (Huis et al., 2010). GADPH and ETIF5A were found to be the most stable, and ETIF5A gene chosen arbitrarily from this pair as the internal control. Thirteen genes were selected for qRT-PCR, as an independent validation of the accuracy of the RNA-Seq results (Figure 2). These genes were selected in part because they were all transcription factors from gene families that could be potentially associated with early differentiation events in the shoot apex including specification of vascular/phloem identity (Zhao et al., 2005; Kalve et al., 2014; De Rybel et al., 2016). Real-time PCR was performed in Applied Biosystems 7500 Fast Real-time PCR System following the manufacturer's protocol. Each amplification reaction was 10 μl and it consisted of 0.4 μM of each primer, 5 μl SYBR Green Master Mix and 2.5 μl 16-fold diluted cDNA. Threshold cycles (CT) were determined through 7500 Fast Software. The PCR program used was as follows: 95°C for 2 min, 40 cycles of 95°C for 10 s and 60°C for 30 s, then 72°C for 30 s and 72°C for 3 min; fluorescence data was collected at 60°C. Data were analyzed using the 2^−ΔΔ^C_T_ method. Primer sequences used are listed in the Supplemental Table 3. As shown in Figure 2, the RNA-seq and qRT-PCR analysis showed highly consistent expression patterns for the 13 genes tested. We therefore conclude that that RNA-Seq data presented here accurately represents differences in transcript expression between the shoot apical region (AR) and the bulk of the stem (BR).

## Author contributions

NZ conducted all experiments, and assisted in analysis and writing of the manuscript. MD designed experiments and assisted in analysis and writing of the manuscript.

## Funding

Genome Canada ABC Program grant TUFGEN; Natural Sciences and Engineering Council (Canada) Discovery Grant 2014-03596 to MD; China Scholarship Council fellowship to NZ.

### Conflict of interest statement

The authors declare that the research was conducted in the absence of any commercial or financial relationships that could be construed as a potential conflict of interest.
